# Special Issue: “Current Trends and Outcomes of Infective Endocarditis”

**DOI:** 10.3390/jcm12154935

**Published:** 2023-07-27

**Authors:** Petros Ioannou, Diamantis P. Kofteridis

**Affiliations:** 1 School of Medicine, University of Crete, 71003 Heraklion, Greece; 2 Internal Medicine Department, University Hospital of Heraklion, 71110 Heraklion, Greece

Infective endocarditis (IE) is an infectious disease involving the endothelium of the heart and, more commonly, the cardiac valves and prosthetic material (such as pacemakers and implantable defibrillators), and carries high morbidity and mortality rates [1,2]. Its epidemiology and microbiology change over time [3,4]. More specifically, the age of diagnosis and the rate of prosthetic material infection are increasing. Moreover, microbiological changes that occur with an increasing rate of enterococcal IE could be associated with the increasing age of patients [3,5]. This Special Issue of the *Journal of Clinical Medicine* (*JCM*) focuses on recent clinical research on IE.

In an original article by Meidrops et al., the clinical characteristics and outcomes of patients with IE requiring cardiac surgery were described [6]. More specifically, the study included 144 adult patients with IE due to *S. aureus*, *Streptococcus* spp., *E. faecalis*, or coagulase-negative staphylococci (CoNS) and an indication for cardiac surgery. *S. aureus*, was the causative agent of IE in 30.6% of cases, *Streptococcus* spp. in 24.3%, *E. faecalis* in 22.9%, and coagulase-negative staphylococci in 22.2%. The presence of a bicuspid aortic valve was the most common predisposing factor and was noted in 23.5% of patients, while intravenous drug use was noted in 11.8% of patients. In-hospital mortality was similar between all of the pathogens; however, patients with IE due to *S. aureus* had worse long-term prognoses. Moreover, IE due to *S. aureus* was associated with higher rates of embolic phenomena. IE due to CoNS was more frequent in cases of prosthetic valve endocarditis, and was also associated with more frequent perivalvular complications.

In patients who present with bloodstream infections (BSI), empirical therapy and the prompt provision of effective treatment are of utmost importance. In their original article, Schumann et al. evaluated the impact of additional FilmArray^®^ Blood Culture Identification Panel (FA BCID 1.0) analysis on the time to optimal antimicrobial treatment, the duration of stay in the intensive care unit (ICU), mortality in the ICU, and the reduction in procalcitonin (PCT) in the ICU of a German tertiary hospital [7]. The FA BCID is able to detect 24 bacterial and fungal microorganisms and three resistance genes in positive blood cultures in 70 min. In total, 179 patients with 200 BSI episodes were enrolled in the prospective-intervention group, while 150 patients with 170 BSI episodes were included in the retrospective control group. In the intervention group, BSI data were evaluated via matrix-assisted laser desorption ionization–time-of-flight mass spectrometry (MALDI-TOF MS) and FA BCID from January 2019 to August 2020, while the data from the control group were retrospectively derived from MALDI-TOF results from 2018. Effective and appropriate antimicrobial treatment was provided earlier in the intervention group, with a median reduction of 17 h compared to the control group. Moreover, necessary changes in the antimicrobial treatment of patients in the intervention group were made a median of 24 h earlier compared to the control group. The length of stay in the ICU, the reduction in PCT levels, and the duration of mechanical ventilation were not significantly different among the two groups. Mortality was similar in both patient groups. These results imply that the implementation of FA BCID can help enable earlier antimicrobial treatment in patients with BSI by providing rapid and accurate microbiologic results and information regarding markers of antimicrobial resistance.

In recent years, the antimicrobial treatment of IE has changed according to the guidelines [8]. Importantly, the treatment of *Enterococcus faecalis* IE has changed due to the introduction of treatment with a combination with ampicillin and ceftriaxone instead of ampicillin and gentamicin [9]. Marino et al. address the issue of adverse events associated with aminoglycosides (namely their ototoxicity and nephrotoxicity), review preclinical evidence for dual beta-lactam therapy, and review data from clinical studies that provide information on dual beta-lactam therapy in patients with IE due to *E. faecalis* [10]. The fact that the success and mortality rates of dual beta-lactam therapy are similar to those of treatment with gentamicin, along with a lower rate of serious adverse events and a lack of need to measure serum drug levels, makes dual beta-lactam treatment a promising option for patients with IE due to *E. faecalis*.

Patients who have undergone solid organ transplantation may, on rare occasions, develop IE, which may exhibit unique characteristics due to the extensive exposure of these patients to healthcare systems, and due to their immunocompromised status. In their systematic review, Ioannou et al. that aimed to systematically review all published cases of IE in patients with liver transplantation and describe their epidemiology, microbiology, clinical characteristics, treatment, and outcomes. PubMed, Scopus, and Cochrane Library were searched until January 2021 for studies that provided such information [11]. In total, 39 studies containing the data of 62 patients were included in their analysis. The most commonly isolated pathogens were Gram-positive microorganisms (69.4%), fungi (25.8%), and Gram-negative microorganisms (9.7%). In 9.3% of cases, IE was culture-negative. The most commonly infected valve was the aortic valve, followed by the mitral, tricuspid, and pulmonary valves. The most commonly used antimicrobials were aminoglycosides, vancomycin, and aminopenicillins, and surgical management was performed in about 50% of patients. A total of 57.4% of the patients were cured, and the overall mortality was 43.5%.

The same research group, in another systematic review, aimed to systematically review all published cases of IE due to *Moraxella catarrhalis*, the most clinically relevant species among *Moraxella* spp. [12]. In a systematic review of PubMed, Scopus, and Cochrane library (through to 8 December 2021), 27 studies that contained epidemiological, clinical, and microbiological data, as well as treatment data and the outcomes of 31 patients with IE due to *Moraxella* spp., were included. A prosthetic valve was present in 25.8% of cases, and the mitral valve was the most commonly infected intracardiac site. The most commonly seen clinical phenomena were fever, sepsis, and embolic phenomena. The treatments most commonly included cephalosporins, aminoglycosides, aminopenicillins, and penicillin, and the overall mortality was 12.9%.
